# Supplementary material for: MetaRibo-Seq measures translation in microbiomes
Source: Nat Commun. 2020 Jun 29;11:3268. doi: 10.1038/s41467-020-17081-z (PMC7324362; doi:10.1038/s41467-020-17081-z)
Supplement: Supplementary file 10 — Supplementary Data 7 [file 41467_2020_17081_MOESM10_ESM.zip › File2/Confidence_High_Taxonomy/199768_out.krona.html]

Javascript must be enabled to view this page.

members
magnitude
magnitudeUnassigned
count
unassigned
taxon
rank

199768\_out

145

superkingdom
145
2

145
1239
phylum

class
186801
145

order
186802
145

141
186803
4
family

SRS014613\_contig\_number\_contig-100\_2058.130987SRS052697\_contig\_number\_contig-100\_4166.262996SRS075773\_contig\_number\_contig-100\_5350.244069SRS148721\_contig\_number\_contig-100\_4086.316722

2
572511
genus

33039
1

SRS024492\_contig\_number\_20193
species

1
2292975
species

SRS077392\_contig\_number\_43140

species

SRS011084\_contig\_number\_contig-100\_558.214298SRS014287\_contig\_number\_16621SRS015794\_contig\_number\_13523SRS024663\_contig\_number\_13260SRS1041139\_contig\_number\_contig-100\_1275.23881SRS147139\_contig\_number\_contig-100\_40.248389SRS149784\_contig\_number\_27680
7
39491


SRS011134\_contig\_number\_44459SRS012273\_contig\_number\_contig-100\_2120.283064SRS013476\_contig\_number\_contig-100\_615.229794SRS015694\_contig\_number\_contig-100\_4752.115625SRS016095\_contig\_number\_contig-100\_5485.176041SRS022071\_contig\_number\_contig-100\_2088.234375SRS023914\_contig\_number\_24920SRS024132\_contig\_number\_contig-100\_2777.298033SRS049959\_contig\_number\_44968SRS076929\_contig\_number\_contig-100\_265.152268SRS1041133\_contig\_number\_25075SRS104693\_contig\_number\_9899SRS144537\_contig\_number\_contig-100\_5307.255629SRS893383\_contig\_number\_36439
species
1898203
14

841
114

SRS014923\_contig\_number\_contig-100\_2407.248884SRS016989\_contig\_number\_contig-100\_1838.103182SRS051031\_contig\_number\_36717SRS056259\_contig\_number\_30089SRS077024\_contig\_number\_contig-100\_3965.83319
genus
5

1263105
24

SRS014459\_contig\_number\_contig-100\_898.236940SRS017433\_contig\_number\_contig-100\_657.114065SRS023346\_contig\_number\_7120SRS024388\_contig\_number\_15106SRS048164\_contig\_number\_contig-100\_877.182347SRS049896\_contig\_number\_34340SRS050925\_contig\_number\_38205SRS064276\_contig\_number\_contig-100\_564.225286SRS078419\_contig\_number\_18087SRS097920\_contig\_number\_contig-100\_385.193165SRS103987\_contig\_number\_contig-100\_5512.191329SRS1055056\_contig\_number\_contig-100\_3505.70105SRS142712\_contig\_number\_3182SRS142890\_contig\_number\_44907SRS142980\_contig\_number\_4901SRS143148\_contig\_number\_21396SRS147139\_contig\_number\_contig-100\_570.248919SRS147377\_contig\_number\_contig-100\_3628.75385SRS147766\_contig\_number\_contig-100\_2408.152766SRS148253\_contig\_number\_contig-100\_1629.101452SRS148784\_contig\_number\_contig-100\_607.168695SRS148817\_contig\_number\_contig-100\_1131.80598SRS893259\_contig\_number\_contig-100\_486.54317SRS893270\_contig\_number\_contig-100\_3866.138299
species

species

SRS013158\_contig\_number\_contig-100\_132.42638SRS013951\_contig\_number\_38579SRS014412\_contig\_number\_13063SRS014855\_contig\_number\_2983SRS014948\_contig\_number\_contig-100\_2842.139134SRS015065\_contig\_number\_contig-100\_241.221667SRS015190\_contig\_number\_17024SRS015264\_contig\_number\_18579SRS015431\_contig\_number\_20008SRS015578\_contig\_number\_19817SRS015579\_contig\_number\_19478SRS015854\_contig\_number\_17167SRS015890\_contig\_number\_17450SRS015960\_contig\_number\_22967SRS017821\_contig\_number\_33841SRS018817\_contig\_number\_17116SRS018984\_contig\_number\_contig-80\_2019.239887SRS019068\_contig\_number\_75513SRS019267\_contig\_number\_4295SRS019397\_contig\_number\_13063SRS019787\_contig\_number\_contig-100\_5975.71310SRS022137\_contig\_number\_9501SRS024435\_contig\_number\_38903SRS024549\_contig\_number\_1457SRS043001\_contig\_number\_11230SRS043411\_contig\_number\_15720SRS043701\_contig\_number\_17788SRS045739\_contig\_number\_4847SRS049900\_contig\_number\_20372SRS050422\_contig\_number\_31550SRS050752\_contig\_number\_31024SRS050801\_contig\_number\_contig-100\_2078.2079SRS052027\_contig\_number\_contig-100\_290.122084SRS053335\_contig\_number\_16742SRS054956\_contig\_number\_16722SRS055017\_contig\_number\_contig-100\_1344.209742SRS056273\_contig\_number\_contig-100\_613.199617SRS056519\_contig\_number\_contig-100\_184.114169SRS062427\_contig\_number\_6998SRS063040\_contig\_number\_18587SRS063518\_contig\_number\_contig-100\_140.232532SRS065504\_contig\_number\_contig-100\_2610.222371SRS074670\_contig\_number\_8883SRS075341\_contig\_number\_contig-100\_692.158657SRS075716\_contig\_number\_4718SRS076756\_contig\_number\_6496SRS077454\_contig\_number\_contig-100\_302.120075SRS077641\_contig\_number\_23861SRS078242\_contig\_number\_contig-100\_15782.135092SRS098061\_contig\_number\_13219SRS098644\_contig\_number\_contig-100\_2920.269183SRS098827\_contig\_number\_contig-100\_1559.92872SRS098881\_contig\_number\_28356SRS1041130\_contig\_number\_4850SRS1041132\_contig\_number\_contig-100\_10462.10463SRS104485\_contig\_number\_3089SRS104975\_contig\_number\_20792SRS105153\_contig\_number\_19628SRS1055049\_contig\_number\_contig-100\_4208.4209SRS142503\_contig\_number\_41357SRS143417\_contig\_number\_16022SRS143722\_contig\_number\_contig-100\_1483.94950SRS143780\_contig\_number\_32844SRS144362\_contig\_number\_37083SRS144714\_contig\_number\_30342SRS145497\_contig\_number\_contig-100\_2822.125021SRS147271\_contig\_number\_contig-100\_3000.247119SRS147919\_contig\_number\_contig-100\_300.121326SRS148091\_contig\_number\_contig-100\_1097.97863SRS148424\_contig\_number\_36799SRS149181\_contig\_number\_25725SRS149244\_contig\_number\_4673SRS149879\_contig\_number\_16918SRS893341\_contig\_number\_contig-100\_3702.96828SRS893378\_contig\_number\_23438SRS971276\_contig\_number\_contig-100\_2334.219790
76
166486


SRS058070\_contig\_number\_28770
species
1262940
1

species

SRS011302\_contig\_number\_18800SRS011405\_contig\_number\_17975SRS016954\_contig\_number\_17287SRS047014\_contig\_number\_28094SRS1055022\_contig\_number\_contig-100\_284.149609SRS143070\_contig\_number\_9939SRS145308\_contig\_number\_4325SRS146764\_contig\_number\_contig-100\_846.202220
8
360807

4
31979
family

genus
1485
4


SRS077231\_contig\_number\_contig-100\_4847.160387SRS1041144\_contig\_number\_contig-100\_18318.18319SRS104311\_contig\_number\_contig-100\_1348.267325SRS144135\_contig\_number\_contig-100\_1404.206148
species
2293035
4
